# Cognitive Decline Trajectories and Time-of-Day Physical Activity Patterns: The ARIC Neurocognitive Study

**DOI:** 10.1093/geroni/igaf122.1952

**Published:** 2025-12-31

**Authors:** Sunan Gao, Ciprian Crainiceanu, Anis Davoudi, Ryan Dougherty, Lacey Etzkorn, Amal Wanigatunga, Adam Spira, Jennifer Schrack

**Affiliations:** Johns Hopkins University, Baltimore, Maryland, United States; Johns Hopkins University, Baltimore, Maryland, United States; Johns Hopkins University, Baltimore, Maryland, United States; Rutgers University, New Brunswick, New Jersey, United States; Johns Hopkins Bloomberg School of Public Health, Baltimore, Maryland, United States; Johns Hopkins Bloomberg School of Public Health, Baltimore, Maryland, United States; Johns Hopkins Bloomberg School of Public Health, Baltimore, Maryland, United States; Johns Hopkins Bloomberg School of Public Health, Baltimore, Maryland, United States

## Abstract

Cognitive decline may influence physical activity (PA) patterns in late life, but how cognitive trajectories differentially impact PA throughout the day remains unexplored. We established cognitive trajectories in 2,140 initially cognitively unimpaired Atherosclerosis Risk in Communities participants (mean age 74.1±4.5 years, 59.9% female) over 7.9±1.7 years of follow-up (visits 5-9). Trajectories were derived using linear mixed-effects models assessing overall cognition, language, memory, and executive function across visits. In 810 participants with subsequent accelerometry data, we examined the association between cognitive trajectories and time-of-day PA patterns measured using wrist-worn devices (visit 9). Function-on-scalar regression revealed time-dependent associations between cognitive trajectories and minute-by-minute PA. After adjustment for demographics, health factors, and self-reported PA, each 10% slower annual cognitive decline was associated with 1.5-2.0% higher midday physical activity (noon: 29 counts/minute higher for overall cognition, 40 for executive function, p < 0.001), with minimal associations during evening/overnight hours (p > 0.05). Executive function decline showed the most temporally extensive association (significant across 11.8 hours/day versus 4.3 hours for language). Multivariable linear regression demonstrated that one percent less decline relative to the median decline rate in overall cognitive function was associated with more favorable PA metrics during daytime hours, particularly 12-6pm (p < 0.001): higher total activity (0.035 SD, SE = 0.007), reduced sedentary time (-0.028 SD, SE = 0.007), and lower activity fragmentation (-0.033 SD, SE = 0.007). All cognitive domains showed similar relationships with afternoon PA patterns. These findings suggest that monitoring afternoon physical activity patterns may provide a non-invasive approach for detection of individuals at risk for cognitive decline in aging populations.

